# F208. COGNITION, POSITIVE SYMPTOMS, AND INTERNET USE FOR MENTAL HEALTH IN PEOPLE WITH PSYCHOSIS

**DOI:** 10.1093/schbul/sby017.739

**Published:** 2018-04-01

**Authors:** Kristi-Ann Villagonzalo, Chelsea Arnold, Fiona Foley, Denny Meyer, John Farhall, Susan Rossell, Neil Thomas

**Affiliations:** 1 Swinburne University of Technology; 2 La Trobe University

## Abstract

**Background:**

People with severe mental illness are increasingly using digital resources for mental health, including social media and online interventions. However, individuals’ ability to engage with or benefit from such resources may be impaired by deficits in cognition and insight, and experiences of psychotic symptoms, including paranoia about cyber-security or motives of others in online social interactions. This study aimed to explore the association between cognition, positive symptoms, and internet use for mental health information in adults with psychosis.

**Methods:**

This study used baseline data collected as part of a broader research program investigating a digital recovery-focused intervention for psychosis. Participants completed a questionnaire on their existing internet use, both in general and for mental health information, and a range of cognitive and functioning measures. Cognitive variables included premorbid IQ, estimated using the Wechsler Test of Adult Reading, and composite scores for processing speed, working memory, and executive functioning. The Positive and Negative Syndrome Scale was also administered, with five items used to examine the relationship between mental health-related internet use and psychopathology: Delusions, Grandiosity, Suspiciousness & Persecution, Unusual Thought Content, and Lack of Judgment & Insight. Logistic regressions were used to identify unique predictors of internet use for mental health information, controlling for age and frequency of general internet use.

**Results:**

179 adults with psychosis (mean age = 39.82 years; range = 18–65; SD = 11.0) took part in this study, of whom 157 (87.7%) were regular internet users. Of these, 107 (68.2%) reported regularly using the internet for mental health information, with 33 (20.9%) doing so daily, 28 (17.7%) weekly, and 46 (29.3%) monthly or less. General websites were most commonly used for this purpose (n = 92; 58.6%), followed by video streaming sites (n = 62; 39.5%), social networking sites (n = 52; 33.2%), and forums (n = 34; 21.7%). When age and frequency of general internet use were controlled for, use of any type of website for mental health information was predicted by lower scores on Grandiosity (Exp(B) = .675, 95% CI = .513, .886, p = .005); mental health-related social media use was significantly predicted by lower estimated premorbid IQ (Exp(B) = .964, 95% CI = .937, .991, p = .010); lower scores on Unusual Thought Content predicted use of both video networking sites (Exp(B) = .629, 95% CI = .403, .981, p = 041) and forums (Exp(B) = .576, 95% CI = .379, .876, p = .010) for mental health information; while use of general websites for mental health information was not uniquely predicted by any cognitive or symptom variables.

**Discussion:**

While internet use for mental health information is now common among people with severe mental illness, the presence of psychotic symptoms may inhibit such information-seeking behaviour, particularly when using interactive websites such as video streaming sites and forums. Cognitive functioning may also affect how online sources of mental health information are selected. However, using general websites for mental health information is common regardless of cognition and symptom severity, with implications for how such resources should be designed.

